# Characterization of Novel MSX1 Mutations Identified in Japanese Patients with Nonsyndromic Tooth Agenesis

**DOI:** 10.1371/journal.pone.0102944

**Published:** 2014-08-07

**Authors:** Seishi Yamaguchi, Junichiro Machida, Munefumi Kamamoto, Masashi Kimura, Akio Shibata, Tadashi Tatematsu, Hitoshi Miyachi, Yujiro Higashi, Peter Jezewski, Atsuo Nakayama, Kazuo Shimozato, Yoshihito Tokita

**Affiliations:** 1 Department of Maxillofacial Surgery, School of Dentistry, Aichi-Gakuin University, Nagoya, Aichi, Japan; 2 Department of Dentistry and Oral Surgery, Aichi Children’s Health and Medical Center, Obu, Aichi, Japan; 3 Department of Perinatology, Institute for Developmental Research, Aichi Human Service Center, Kasugai, Aichi, Japan; 4 Department of Embryology, Institute for Developmental Research, Aichi Human Service Center, Kasugai, Aichi, Japan; 5 Department of Oral and Maxillofacial Surgery, Japanese Red Cross Society Himeji Hospital, Himeji, Hyogo, Japan; 6 Department of Periodontology, University of Alabama at Birmingham School of Dentistry, Institute of Oral Health Research, Birmingham, Alabama, United States of America; 7 Department of Oral and Maxillofacial Surgery, Toyota Memorial Hospital, Toyota, Aichi, Japan; National Cancer Institute, National Institutes of Health, United States of America

## Abstract

Since *MSX1* and *PAX9* are linked to the pathogenesis of nonsyndromic tooth agenesis, we performed detailed mutational analysis of these two genes sampled from Japanese patients. We identified two novel *MSX1* variants with an amino acid substitution within the homeodomain; Thr174Ile (T174I) from a sporadic hypodontia case and Leu205Arg (L205R) from a familial oligodontia case. Both the Thr174 and Leu205 residues in the MSX1 homeodomain are highly conserved among different species. To define possible roles of mutations at these amino acids in the pathogenesis of nonsyndromic tooth agenesis, we performed several functional analyses. It has been demonstrated that MSX1 plays a pivotal role in hard tissue development as a suppressor for mesenchymal cell differentiation. To evaluate the suppression activity of the variants in mesenchymal cells, we used the *myoD*-promoter, which is one of convenient reporter assay system for MSX1. Although the gene products of these *MSX1* variants are stable and capable of normal nuclear localization, they do not suppress *myoD-*promoter activity in differentiated C2C12 cells. To clarify the molecular mechanisms underlying our results, we performed further analyses including electrophoretic mobility shift assays, and co-immunoprecipitation assays to survey the molecular interactions between the mutant MSX1 proteins and the oligonucleotide DNA with MSX1 consensus binding motif or EZH2 methyltransferase. Since EZH2 is reported to interact with MSX1 and regulate MSX1 mediated gene suppression, we hypothesized that the T174I and L205R substitutions would impair this interaction. We conclude from the results of our experiments that the DNA binding ability of MSX1 is abolished by these two amino acid substitutions. This illustrates a causative role of the T174I and L205R MSX1 homeodomain mutations in tooth agenesis, and suggests that they may influence cell proliferation and differentiation resulting in lesser tooth germ formation *in vivo*.

## Introduction

Coding mutations within *MSX1* and *PAX9* have been identified in patients with either nonsyndromic or syndromic forms of tooth agenesis [1]–[9]. Based on the number of missing permanent teeth, excluding the third molars, tooth agenesis is classified into three categories. Hypodontia is generally defined as agenesis with the absence of less than six teeth, oligodontia is a condition in which six or more teeth are missing, and anodontia is the term used for complete tooth loss. Both in vivo and in vitro studies have revealed that Msx1 and Pax9 are essential for the induction of bone morphogenetic protein 4 (Bmp4), which is regarded as the pivotal step in mammalian tooth development [10]. Whilst the induction of BMP4 is considered to play the principal role in tooth germ development, repression of mesenchymal cell differentiation mediated by MSX1 has also been demonstrated to be a pivotal step for early development of hard tissues [12]. In addition, it is noteworthy that MSX1 by itself, unlike PAX9, cannot activate the Bmp4-promoter in vitro, but can potentiate PAX9 activity as a synergic cofactor. The molecular mechanism of human tooth agenesis mediated by MSX1 mutation is however independent of PAX9, since most MSX1 mutations related to oligodontia can potentiate PAX9-induced Bmp4-promoter activation [11]. Furthermore, it has been reported that MSX1 reduces the expression level of several genes that promote mesenchymal cell differentiation both in vitro [13], and in vivo [12]. Hence, to evaluate the ability of transcriptional gene repression of MSX1 mutation, we selected *MyoD*-promoter since it is a well-characterized system to assess the MSX1 mediated gene suppression activity [14]–[16]. In addition to this, it has been demonstrated previously that the molecular interaction between Msx1 and Enhancer of Zeste 2 (EZH2) histone H3 methyltransferase, one of the components of PRC2 (polycomb repressive complex 2), is required to suppress mesenchymal cell differentiation both in vitro and in vivo [12]. According to that report, EZH2 mediates gene suppression via the enrichment of tri-methylated histone H3 at lysine 27 on MSX1 genomic binding sites and ectopic myoblast cell differentiation is observed in the limb bones of MSX1-deficient mice [12]. The evidence to date thus suggests that the role of MSX1 is to prevent cell differentiation, and to maintain dental mesenchymal cells in a proliferative state to ensure robust tooth germ formation. We therefore hypothesize that EZH2 is the central player in the downstream cascade of MSX1 during the early development of human hard tissue, including permanent teeth.

In our current study, we describe two novel variants of the *MSX1* gene identified in two Japanese patients with isolated tooth agenesis. Since both of these variants involved amino acid substitutions in the MH4/homeodomain of MSX1, we performed functional analyses to confirm their pathogenicity. We conclude from our analysis that these amino acid substitutions are etiologic because they impair the transcriptional repression of MSX1 due to a loss of the DNA binding ability of the mutant proteins, whilst exhibiting no differences from wild-type MSX1 in terms of nuclear localization and molecular interactions with EZH2.

## Subjects and Methods

### Patients

This study was approved by the Committee on the Ethics of Human Experimentation, Aichi-Gakuin University, and the Institute for Developmental Research. A blood or hair sample was obtained from the participants with written informed consent. Well-characterized Japanese tooth agenesis patients were investigated in this study (four oligodontia cases involving the absence of six or more permanent teeth excluding the third molars; and 15 hypodontia cases with an absence of one to five permanent teeth excluding the third molars). The diagnoses of all patients were based on X-rays or study models. The control group comprised 100 Japanese samples from unrelated individuals who had no family history of tooth agenesis and/or facial malformation.

### Sequencing and Mutation Analysis of Candidate Genes

All exons of the *MSX1* and *PAX9* genes including the splice sites were sequenced. The primers for *MSX1* and *PAX9* have been described previously [6], [17], [18]. The sizes of the amplified DNA fragments were as follows: *MSX1* exon1, 766 bp; exon2, 698 bp; *PAX9* exon1, 273 bp; exon2, 695 bp; exon3, 264 bp; exon4, 450 bp. The PCR products were sequenced using the BigDye Terminator v1.1 Cycle Sequencing Kit (Applied Biosystems, Foster City, CA) as previously described [19].

### Plasmids

To construct the variant MSX1 expression vectors, *in vitro* site-directed mutagenesis was performed with FLAG-tagged human wild-type MSX1 [6] as a template. The amplified DNA fragments were subcloned into pcDNA3 (Invitrogen, San Diego, CA). The *myoD*-reporter plasmid (6.9 kb of DNA fragment of *myoD*-promoter region-driven firefly luciferase) [20] and myc-EZH2 plasmid [21] were kindly provided by Dr. Cory Abate-Shen (Columbia University) and Dr. Akiko Murayama (University of Tsukuba), respectively.

### 3D Models of MSX1 Variants

Since the MH4/homeodomain protein sequence in MSX1 is conserved between human and mouse, 3D structural data of the mouse Msx1-DNA molecular complex was retrieved from the NCBI Brookhaven Protein Data Bank (PDB) database (PDB ID, 1IG7) and used as a modeling template. The 3D models of the MSX1 variants were generated using Waals software ver. 1.1.2. (Altif Laboratories Inc., Tokyo, Japan) based on crystal structure.

### Culture conditions for C2C12 Cell Differentiation

The mouse C2C12 cell line (ATCC CRL-1772; American Type Culture Collection, Manassas, VA) can differentiate into muscle, adipocyte, or osteoblastic lineages [22]–[24]. According to previous reports, these cells are maintained in Dulbecco’s modified Eagle’s medium (DMEM; Sigma) containing 20% fetal bovine serum. Differentiation was induced using differentiation medium containing DMEM and 2% horse serum.

### DNA Transfection and Luciferase Reporter Assays

DNA transfections and luciferase assays were performed according to previously reported methods with minor modifications [6], [25]. Briefly, transfection experiments were carried out in mouse C2C12 cells at 40–50% confluence using Lipofectamine 2000 (Invitrogen) in accordance with the manufacturer’s instructions. Each transfection was done in triplicate in a gelatin coated 24-well plate (Iwaki glass, Tokyo, Japan) with 2.0 µg of *myoD*-firefly luciferase reporter vector, 3–20 ng of MSX1 expression vectors, and 2 ng of pRL-TK, an expression vector for sea pansy (*Renilla reniformis*) luciferase used as an internal control. Cells were incubated for 72 hours after transfection. Firefly luciferase and Renilla luciferase activities were measured in a luminometer using the Dual Luciferase Reporter assay system kit (Promega, Madison, WI). Cells were lysed in 100 µl of passive lysis buffer with agitation for 30 minutes at room temperature and 10 µl aliquots were used for measurements. Luciferase activity elicited by the *myoD*-promoter constructs was normalized for variations in transfection efficiency using Renilla luciferase as an internal standard.

### Western Blotting

Western blotting was done as described previously [26] with minor modifications. Briefly, HEK293 transfectants were extracted with lysis buffer (1% Triton X-100, 1 mM EDTA in Tris-buffered saline; TBS) and these lysates were subjected to SDS-PAGE (12% gel), and transferred to Immobilon-P membranes (Millipore Corporation, Bedford, MA). The membranes were probed with an anti-FLAG M2 monoclonal antibody (1∶2000; Sigma, St. Louis, MO), and signals on the blots were detected with a horseradish peroxidase-conjugated secondary antibody and ECL2 reagent (Amersham Life Science, Cleveland, OH).

### Immunoprecipitation

HEK293 cells were cultured in DMEM supplemented with 10% FBS and transfected with FLAG-MSX1 and myc-EZH2 expression vectors. The cells were then treated with lysis buffer (10 mM Tris, pH 7.4, 150 mM NaCl, 1 mM EDTA, 0.1 mM dithiothreitol, 1% Triton X-100). MSX1 variants were immunoprecipitated from the lysates with anti-FLAG M2 monoclonal antibody and protein G sepharose beads (GE Healthcare Life Sciences, Piscataway, NJ). After washing of the beads, the samples were run on a 9% SDS-PAGE gel and analyzed by Western blotting with an anti-myc antibody.

### Immunolocalization of Variant MSX1 Proteins in Mammalian Cells

To demonstrate the *in vivo* expression of wild-type and mutant MSX1, HEK293 cells were transfected with expression vectors for these products using Lipofectamine 2000 (Invitrogen, Grand Island, NY) as described previously [6]. Forty-eight hours post-transfection, the cells were fixed with 4% paraformaldehyde/TBS and permeabilized with 1% Triton X-100/TBS prior to incubation with an anti-FLAG M2 monoclonal antibody (1∶1000) for 60 minutes at 37°C. The cells were then incubated for 60 minutes at room temperature with DAPI (1 µg/ml), Alexa-488 phalloidin (2 U/ml), and Cy3-conjugated goat anti-mouse antibody (Jackson ImmunoResearch Laboratories Inc., West Grove, PA, 1∶1000), respectively, in PBS. After washing of the cells with PBS three times, immunostaining signals were visualized under an Olympus BH-2 microscope.

### Electrophoretic Mobility Shift Assay (EMSA)

EMSA was performed as described previously [21]. Briefly, complementary oligonucleotides harboring the MSX1 binding sequence (5′-CATGACCCCAATTAGTCCTGGCAG-3′ and 5′-gtgCTGCCAGGACTAATTGGGGTCATG-3′) were annealed and labeled with [α-^32^P]dCTP [27]. The labeled probe at volumes corresponding to 10,000–20,000 cpm was incubated with 5 µl of cell lysates (prepared as described above) in a total volume of 15 µl (10 mM HEPES, 50 mM KCl, 0.1 mM EDTA, 1 mM dithiothreitol, 10 mM MgCl, and 10% glycerol), containing 1 µg of poly(dI-dC) for 15 minutes at room temperature. The reactions were analyzed by non-denaturing 4% polyacrylamide gel electrophoresis. For supershift assays, 2 µg of anti-FLAG M2 monoclonal antibody was added to the binding reaction.

## Results

### Pedigree and Diagnosis

**Figure 1:**
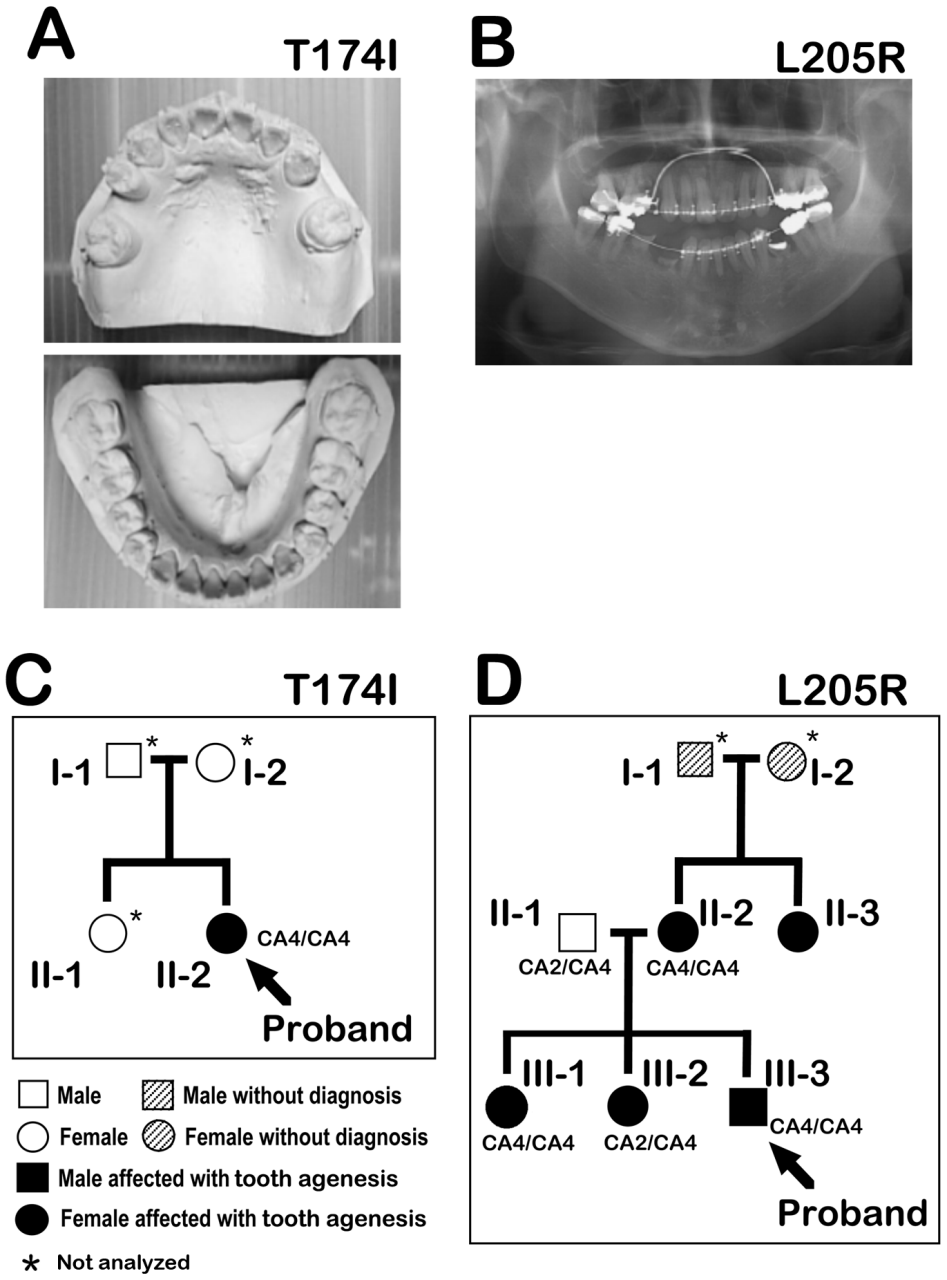
Pedigree analysis of tooth agenesis. **A:** Plaster cast of the maxillary (upper panel), and mandibular (lower panel) of the T174I proband exhibiting five missing teeth. **B:** Panoramic radiograph of the L205R proband with nine missing teeth. **C and D:** Pedigree for the tooth agenesis family of both the T174I (C), and L205R (D) probands. Darkened symbols represent affected, clear symbols indicate normal unaffected, hatched symbols indicate unable to diagnose caused by acquired tooth loss, squares indicate males, and circles indicate females. A type of CA-repeat polymorphism in the *MSX1* intron is indicated i.e. CA1 (12×CAs), CA2 (11×CAs), CA3 (10×CAs), CA4 (9×CAs), CA5 (8×CAs). Asterisks indicate family members that were not analyzed.

We analyzed the *MSX1* and *PAX9* nucleotide sequences of 19 Japanese tooth agenesis patients, and thereby identified two novel mutations in the *MSX1* gene (Fig. 1, 2A, B). The first case harboring a novel *MSX1* variant was an apparent sporadic form of hypodontia in a 23-year-old female proband involving five missing teeth (plus the absence of an additional four third molars for a total of nine missing teeth; Fig. 1A, C). Unfortunately, we were forced to exclude the other members of her family from this study as we could not obtain informed consent. There was, however, no family history of congenitally missing teeth (assessed by medical interview). The second case was determined to be a familial case of oligodontia, wherein five of eight family members were affected in the pedigree through three generations (Fig. 1B, D). The proband (III-3) was a 26-year-old male missing 9 teeth (plus an additional four missing third molars for a total of 13 missing teeth). His two sisters (III-1 and III-2) also exhibited an oligodontia phenotype (Table 1). No orofacial cleft or other craniofacial abnormalities and no *PAX9* gene variations were present in any members of the families of these two cases.

**Figure 2:**
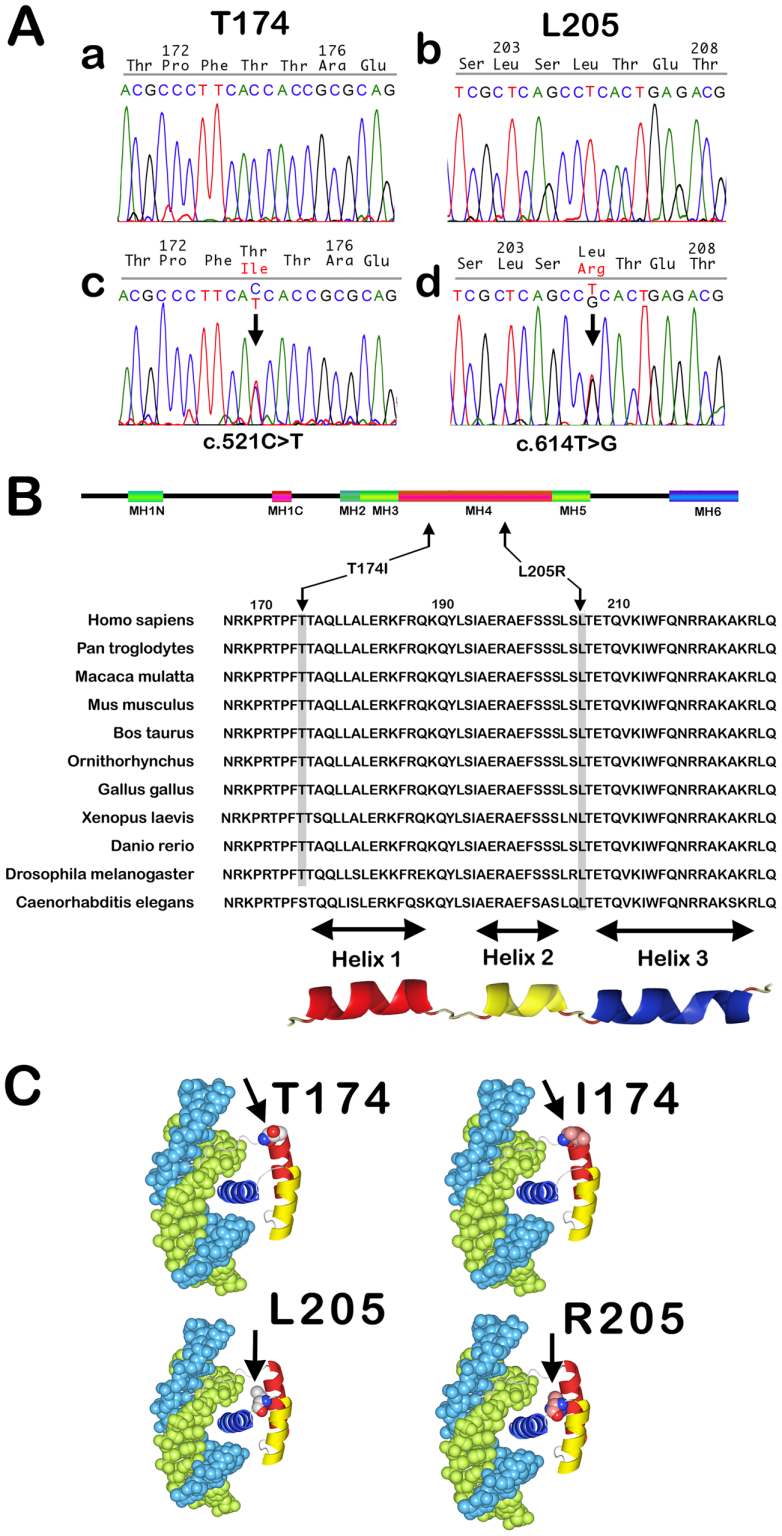
MSX1 variants isolated from tooth agenesis patients. **A:** DNA sequences of MSX1 exon 2 in unaffected (a and b) and affected (c and d) individuals. The C-T transition at position 521 (T174I), and T-G transition in position 614 (L205R) in the mutant sequences cause amino acid substitutions within the MH4/homeodomain of MSX1. **B:** Schematic representation of the human MSX1 protein. Multiple sequence alignments of the MSX1 MH4/homeodomain sequences sorted by species and homology are shown. Sequences were obtained from the SwissProt database and thereby aligned. Conservation of the key Thr174 and Leu205 amino acids (black arrows) across species is indicated by gray shading. The leucine at position 205 is universally conserved at that position but the threonine at position 174 is replaced by serine, which has a similar polarity, in *C. elegans* Msx1 (See Additional file 1 in [43]). **C:** 3D models of mutant and wild-type MSX1 based on the crystal structures of the Msx1 MH4/homeodomain in complex with DNA (PDB ID 1IG7).

**Table 1:** Summary of Tooth Agenesis Phenotypes in the L205R and T174I families.

Pedigree ID		*Congenitally Missing Permanent Teeth																
Dental Arch		Right								Left								
**A**	**T174I**	**8**	**7**	**6**	**5**	**4**	**3**	**2**	**1**	**1**	**2**	**3**	**4**	**5**	**6**	**7**	**8**	
	**II-2:**																	MSX1- c.521C>T T174I(heterozygous)
	Maxillary	*	*		*								*	*		*	*	
	Mandibular	*															*	
**B**	**L205R**	**8**	**7**	**6**	**5**	**4**	**3**	**2**	**1**	**1**	**2**	**3**	**4**	**5**	**6**	**7**	**8**	
	**II-1**																	no mutations in MSX1
	Maxillary	no congenitally missing teeth																
	Mandibular																	
	**II-2**				hypodontia, but it’s unknown which teeth are congenitally missing													MSX1- c.614T>G L205R(heterozygous)
	Maxillary																	
	Mandibular																	
	**III-1**																	MSX1- c.614T>G L205R(heterozygous)
	Maxillary	?	*	*		*			*	*	*		*		*		?	
	Mandibular	?		*	*	*	*		*	*		*	*	*	*		?	
	**III-2**																	MSX1- c.614T>G L205R(heterozygous)
	Maxillary	*			*			*				*		*			*	
	Mandibular	*			*	*							*	*			*	
	**III-3:**																	MSX1- c.614T>G L205R(heterozygous)
	Maxillary	*			*	*							*	*			*	
	Mandibular	*			*	*		*					*	*			*	

1, central incisor; 2, lateral incisor; 3, canine; 4 and 5, first and second premolars, respectively; and 6–8, first, second, and third molars, respectively.

In another pedigree investigated in this study, we identified one sporadic hypodontia case with a unilateral cleft lip and palate, but no *MSX1* or *PAX9* gene variations were detected. All the mothers of the probands had neither been smokers nor habitual drinkers. All of the primary teeth of these study participants were reported to be normal in size, shape, and number.

### Mutation Search in Tooth Agenesis Patients

We analyzed the entire coding regions of the *MSX1* and *PAX9* genes. Two novel single-base substitutions were identified in the *MSX1* gene of two unrelated patients: a C to T transition at nucleotide 521 (C521T, T-allele: ACGCCCTTCA***C/T***CACCGCGCAG), and a T to G transition at nucleotide 614 (T614G, G-allele: TCGCTCAGCC***T/G***CACTGAGACG), and not in the unaffected members of the corresponding families (Fig. 2A). These variants had not been previously recorded in the 1000 Genomes Project database, dbSNP 137 database, or UCSC browser database. In addition, these variants were not evident in 200 normal Japanese control chromosomes (not shown), nor among 500 other population control individuals as described in our previous report [18], giving a global allele frequency estimation of <.001. The C to T transversion results in a replacement of threonine with isoleucine at position 174 (T174I), and the T to G transversion a substitution of the leucine residue at position 205 with arginine (L205R), within the MH4/homeodomain region of *MSX1* (Fig. 2B, C).

### *MyoD*-Promoter Repression Activity of MSX1 Variants in vitro

**Figure 3:**
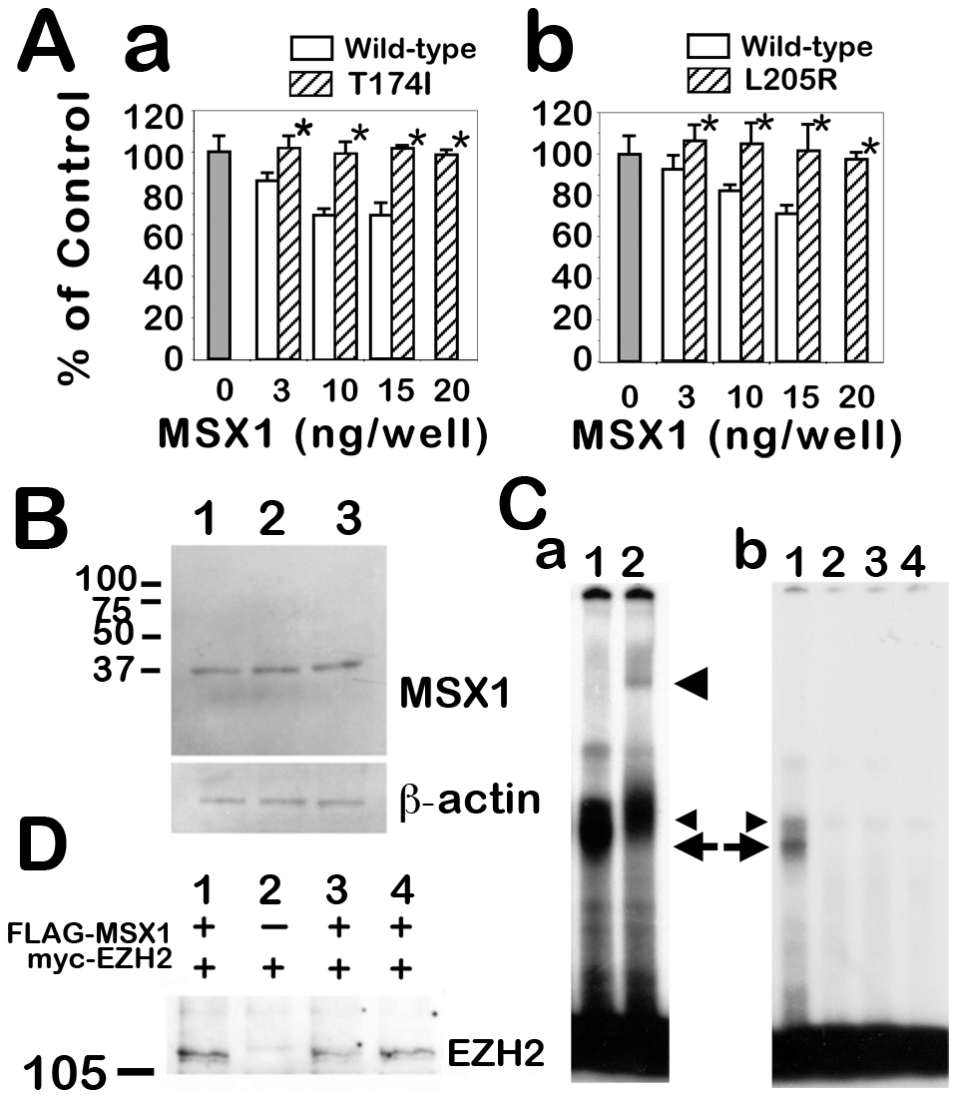
Functional analysis of tooth agenesis-causing MSX1 mutations. **A:** *myoD*-promoter repression activity of T174I and L205R variants in C2C12 cells. The C2C12 cells were cotransfected with an MSX1 expression vector, the firefly luciferase reporter gene driven by the *myoD*-promoter and sea pansy luciferase reporter gene driven by the thymidine kinase promoter. After differentiation treatments, firefly luciferase activity was measured and normalized with respect to the sea pansy luciferase activity. Open bars indicate human wild-type MSX1 transfectant data (3–15 ng/well); hatched bars denote data from experiments utilizing transfectants of the T174I (a) and L205R (b) variants of MSX1 (3–20 ng/well), respectively. The relative luciferase activity is expressed as a percentage of the negative control (gray bars). Each transfection point was carried out in triplicate in a gelatin-coated 24-well plate. The experiments were performed three times and representative data are shown. Error bars indicate standard deviation. Asterisks indicate no significant differences against the control (P-value >0.1). **B:** Stability and expression level of wild-type and mutant proteins. Western blot analysis was performed using extracts prepared from HEK293 cells transfected with FLAG-wild-type-MSX1 (lane 1), FLAG-T174I-MSX1 (lane 2) or FLAG-L205R-MSX1 (lane 3). These whole-cell extracts were immunoblotted with an anti-FLAG antibody (upper panel) and anti-beta actin antibody as an internal standard (lower panel). **C:** Electrophoretic mobility shift assay of MSX1. **a:** A double-stranded oligonucleotide probe containing a consensus binding-site for MSX1 interacts with proteins in the wild-type MSX1 transfectant cell lysate (lane 1, arrow and small arrowhead). The DNA binding ability of MSX1 is diminished by the T174I and L205R substitutions. Wild-type-MSX1 protein and oligo DNA complex observed in lane 1 (arrow) was not detected in the MSX1 variant transfectant lysates (lane 2, T174I-MSX1; lane 3, L205R-MSX1), or HEK293 parental cells (lane 4, negative control). **b:** Supershift experiments using an anti-FLAG antibody. The binding specificity was confirmed by a super-shift induction with anti-FLAG antibody (lane 2). A protein-DNA-antibody complex formed (large arrowhead), and the band indicated by the arrow disappeared. This indicates that this molecular complex denoted by the arrow contained FLAG-tagged wild-type MSX1 protein, and that the protein indicated by the small arrowhead is an unknown product that bound to the probe. **D:** Molecular interaction between MSX1 and EZH2 histone methyltransferase. Myc-EZH2 protein was coimmunoprecipitated with FLAG-MSX1 (lane 1). EZH2 protein was not detected in the MSX1-null EZH2 transfectant HEK293 cell lysate (lane 2). The MSX1-EZH2 interaction was observed in the FLAG-T174I-MSX1 sample (lane 3), and in the FLAG-L205R-MSX1 sample (lane 4).

It has been reported that MSX1 suppresses *myoD*-promoter activity and maintains cell proliferation during mesenchymal cell differentiation. Thus, the transcriptional regulation activities of the novel MSX1 variants in our current study were measured in a mesenchymal cell line, C2C12, transfected with a luciferase-reporter vector containing the *myoD*-promoter. A considerable level of luciferase activity was measured in differentiated C2C12 cells in this assay. In addition, whereas the reporter activity was remarkably reduced in the wild-type MSX1 transfected cells in a dose-dependent manner, no repression activity was detectable in the MSX1 variant-transfected cells (Fig. 3Aa, b). However, the stability of these variant proteins appeared to be unaffected by the amino acid substitutions since we could not detect any significant differences in the protein expression level between wild-type MSX1 and the T174I or L205R mutant proteins (Fig. 3B).

### DNA Binding Ability of the MSX1 variants

Electrophoretic mobility shift assay (EMSA) analyses showed that the DNA-binding ability of the MSX1 protein was abolished by either the T174I or L205R mutations. Since there were two molecular complexes detected by a DNA probe containing the MSX1 consensus binding site (indicated by the small arrowhead and arrow in Fig. 3Ca, lane 1), we performed an additional supershift assay with an anti-FLAG antibody to confirm that these were FLAG-MSX1 containing complexes. The results showed that one of the molecular complexes indeed contained FLAG-MSX1, as previously reported [27], because the FLAG-MSX1/DNA complex (indicated by the arrow) was shifted to a higher molecular weight following incubation with an anti-FLAG antibody (Fig. 3Cb, lane 2, large arrowhead). The protein indicated by the small arrowhead is therefore an unknown endogenous protein in the HEK293 cells. In contrast to the wild-type MSX1 protein (Fig. 3Ca, lane 1, arrow), the mutant T174I or L205R MSX1 variants showed an absence of DNA binding activity (Fig. 3Ca, lanes 2–4).

### Molecular Interactions between the MSX1 Variants and EZH2

Since the C-terminus incorporating the MH4/homeodomain of MSX1 is responsible for the interaction with EZH2 [12], we investigated whether the T174I or L205R substitutions could disrupt this complex formation. However, no significant differences were detected between wild-type and T174I or L205R variants with respect to molecular complex formation with the EZH2 protein (Fig. 3D).

### Nuclear Localization of the T174I or L205R MSX1 proteins

**Figure 4:**
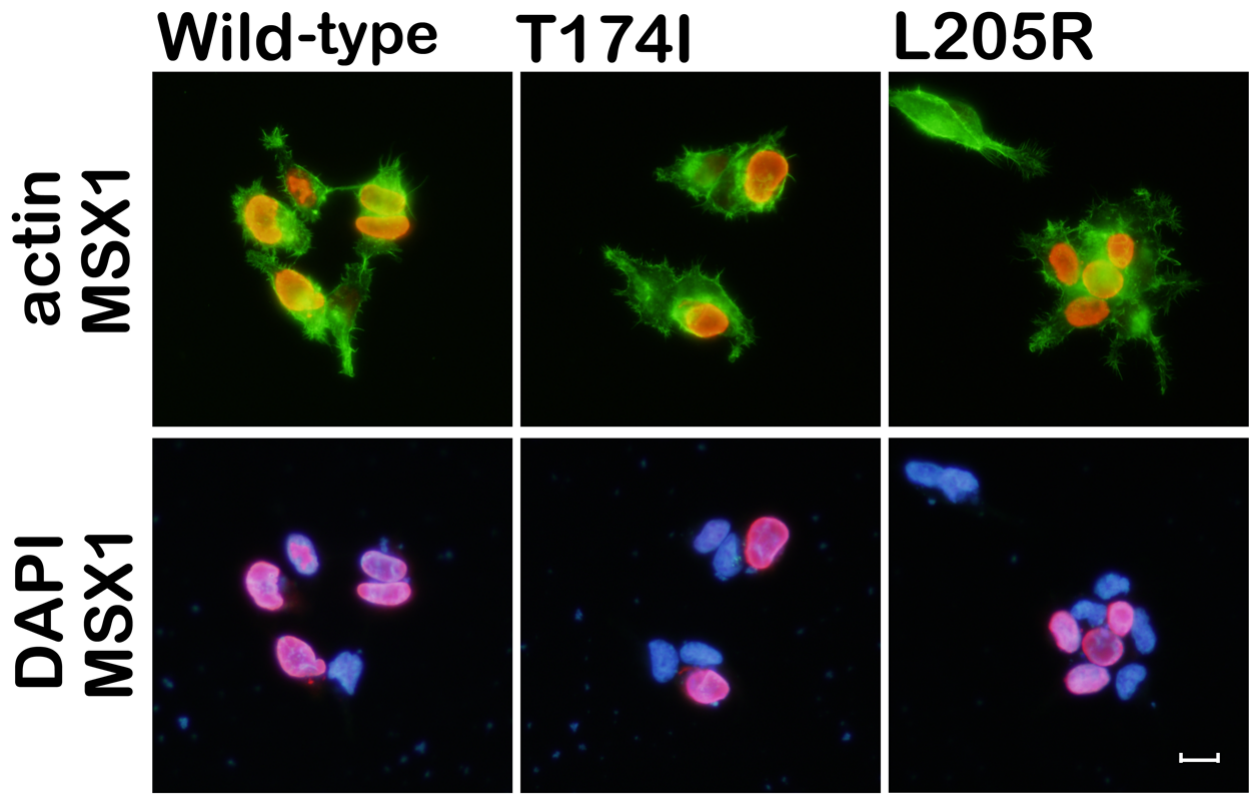
Nuclear localization of MSX1 variants. Immunolocalizations of FLAG-tagged wild-type, T174I, and L205R MSX1 products were similar when these variants were expressed in HEK293 cells. MSX1 (FLAG, red signal); nuclei (DAPI, blue signal). Cell morphologies were visualized by F-actin staining (Phalloidin, green signal). Neither the T174I nor L205R mutation affects the nuclear localization of MSX1 in vitro. Scale bar 20 µm.

The homeodomain of MSX1 is also responsible for the nuclear localization of this protein [11]. Thus, we investigated whether the T174I or L205R mutations had any effect on this process. HEK293 cells were transfected with plasmids encoding FLAG-tagged wild-type, T174I or L205R MSX1. By immunocytochemistry, FLAG signals were equally localized in the nucleus in all transfectants at 48 hours post-transfection (Fig. 4).

### CA-repeat in the MSX1 Intron

It has been suggested that the tooth phenotypes may be a sub-phenotype of oral-facial clefting disorders [28]. The intronic CA-repeat (CA4) has been shown to be associated with oral-facial clefting [29], [30], whereas the CA2 allele is known to be significantly undertransmitted in a biallelic model of oral facial clefting [31]–[33], and may be protective against that phenotype. Although it is conceivable that the complex disease alleles marked by these CA-repeats could potentially influence the tooth phenotype, our DNA sequencing revealed that the CA-repeat genotype of the T174I proband was unexpectedly homozygous for the CA4 allele (9×CAs; Fig. 1C). Moreover, a heterozygous CA2 allele (11×CAs) was found in one of the affected family members in the L205R pedigree (Fig. 1D; III-2).

## Discussion

We have found in our current study that MSX1 mutations in a familial-form of oligodontia (in a proband with a 13 tooth-loss including four third molars), and in an apparently sporadic-form of hypodontia (a proband with a nine tooth-loss including four third molars; Fig. 1). These mutations are contained within the beginning of the 1st helix of the MH4/homeodomain (T174I) and at the loop domain between the 2^nd^ and 3^rd^ helix in the MH4/homeodomain (L205R), which are highly conserved regions among various species (Fig. 2B, C). We show from reporter analysis using the *myoD*-promoter that both amino acid substitutions affect the transcriptional suppression activity of MSX1, which is one of key molecular mechanisms in mesenchymal cell differentiation (Fig. 3Aa, b). Our EMSA results indicate that the normal MSX1 DNA binding ability is impaired by the T174I and L205R mutations (Fig. 3C). Cory and colleagues have described in their previous report that the EZH2 protein binds to the C-terminus of MSX1 that incorporates its homeodomain [12]. We thus investigated whether these mutations affected the EZH2 binding ability of MSX1. Although we found that these variants can still interact with EZH2 (Fig. 3D) and show a normal subcellular-localization (Fig. 4), the loss of DNA binding ability in these mutant proteins would likely affect the recruitment of EZH2 to the MSX1 target genes that relate to mesenchymal cell differentiation, a process which needs to be suppressed for tooth germ formation.

The MH4/homeodomain of MSX1 is a DNA binding domain that assembles into a three-helical chain fold. The N-terminal arm of this domain is composed of nine amino acid residues, and the 2^nd^ and 3^rd^ helices are arranged in a helix-turn-helix motif (Fig. 2B). Crystallographic studies of the MSX1 homeodomain in complex with DNA have revealed a general model of DNA recognition by homeoproteins [34]–[36]. According to these studies, homeodomains contact their target DNA regions in the major and minor groove using their third helix and N-terminal arm, respectively. In the case of MSX1, the T174 residue contacts the minor groove of the DNA as a part of the N-terminal arm [37] (Fig. 2C). On the other hand, the polypeptide segment Ser204-Leu205-Ser206-Leu207 forms a type IV beta-bend in the predicted structure of the 2^nd^ and 3^rd^ helix of the MH4/homeodomain [38]. Thus, an amino acid substitution at this segment may affect the bend angle between the 2^nd^ and 3^rd^ helices. It is therefore plausible that the T174I and L205R substitutions directly impair DNA binding by MSX1. We were unable to observe any influence of the CA association markers within the families that we evaluated in our current analyses, since a significant correlation between the CA-repeat mutations and tooth phenotypes was not observed in these pedigrees (Fig. 1C, D).

Since our T174I proband was classified as a case of hypodontia based on well-established standards, we had expected that the T174I substitution would partially diminish the suppression activity of MSX1 against the *myoD*-promoter in a manner similar to another MSX1 variant that we previously described [6]. The *myoD*-promoter suppression activity was, however, found to be similar to that of the L205R variant (Fig. 3A), which causes a more severe phenotype (Table 1). There are two possible explanations for this finding. First, it has been reported that the R196P MSX1 mutant isolated from an oligodontia pedigree [1] loses its ability to suppress another MSX1 target, WIP-element [39], which is a regulatory element for restricted expression of Wnt-1 protein in the embryonic mouse forebrain [40]. In the R196P pedigree, the numbers of missing teeth range from 4 to 13. Therefore, it is possible that the T174I substitution may also relate to a more severe tooth agenesis phenotype, and such variable expressivity may result from varying genetic backgrounds associated with the risk of nonsyndromic tooth agenesis [41], [42]. Second, there may be differences between the MSX1 transcriptional regulation that occurs at the *myoD*-promoter and at other promoters related to odontoblast differentiation such as bmp2, bmp4, and runx2, which have been previously shown to be suppressed by MSX1 [13].

Further studies are needed to evaluate MSX1 variants related to tooth agenesis including a deficiency in the regulatory activity of histone H3 tri-methylation. In any case, we show from our current analysis that Thr174 and Leu205 are both crucial for a properly functioning MSX1 MH4/homeodomain structure and robust odontogenesis.
